# Acupuncture Interventions for Alzheimer's Disease and Vascular Cognitive Disorders: A Review of Mechanisms

**DOI:** 10.1155/2022/6080282

**Published:** 2022-09-28

**Authors:** Kunrui Du, Shaojie Yang, Jingji Wang, Guoqi Zhu

**Affiliations:** ^1^Graduate School, Anhui University of Chinese Medicine, Hefei 230012, China; ^2^Anhui Acupuncture and Moxibustion Clinical Medicine Research Center, The Second Affiliation Hospital of Anhui University of Chinese Medicine, Hefei 230061, China; ^3^Key Laboratory of Xin'an Medicine, The Ministry of Education and Key Laboratory of Molecular Biology (Brain Diseases), Anhui University of Chinese Medicine, Hefei 230012, China

## Abstract

Cognitive impairment (CI) related to Alzheimer's disease (AD) and vascular cognitive disorders (VCDs) has become a key problem worldwide. Importantly, CI is a neuropsychiatric abnormality mainly characterized by learning and memory impairments. The hippocampus is an important brain region controlling learning and memory. Recent studies have highlighted the effects of acupuncture on memory deficits in AD and VCDs. By reviewing the literature published on this topic in the past five years, the present study intends to summarize the effects of acupuncture on memory impairment in AD and VCDs. Focusing on hippocampal synaptic plasticity, we reviewed the mechanisms underlying the effects of acupuncture on memory impairments through regulation of synaptic proteins, AD characteristic proteins, intestinal microbiota, neuroinflammation, microRNA expression, orexin system, energy metabolism, etc., suggesting that hippocampal synaptic plasticity may be the common as well as the core link underlying the above mechanisms. We also discussed the potential strategies to improve the effect of acupuncture. Additionally, the effects of acupuncture on synaptic plasticity through the regulation of vascular–glia–neuron unit were further discussed.

## 1. Introduction

With the advent of an aging society and the increasing incidence of cerebral blood disease, aging-related cognitive impairment (CI) has become a key problem worldwide. Alzheimer's disease (AD) and vascular cognitive disorders (VCDs) are two of the major diseases that seriously affect the quality of life of the middle-aged and elderly. CI is caused by age-related, genetic, and environmental factors, including impairments in executive function, attention, visuospatial memory, language and logical ability, emotional control, social behavior, and motivation [1]. A hallmark of the early phase of AD is vessel impairment [2]. AD and VCDs coexist and share similar biomarkers [3], indicating the importance of investigating their mechanisms and identifying treatments for these two types of dementia. In fact, memory impairment is thought to be the core feature of CI, and the mechanisms underlying different processes of memory have attracted much attention in this regard. Recent investigations have identified the mechanisms of learning and memory from the perspectives of neural circuits and physiological and molecular changes [4, 5].

Synaptic plasticity refers to the adaptive changes in the strength or efficacy of synapses between neurons when they undergo various environmental changes, and is one of the most critical characteristics of humans and animals. Synaptic plasticity in the hippocampal CA1 region is closely related to learning and memory [6]. In addition to playing an important role in the development and formation of neural circuits, synaptic plasticity is also closely related to the development of various neurogenic and psychogenic diseases [7–9]. Impairment of hippocampal synaptic plasticity in CI, including AD and VCDs, has been extensively reported [10, 11]. A series of pathologic factors, including abnormal expression of synaptic proteins, inflammatory responses, and toxic releases caused by flora disorder can damage the structural and functional synaptic plasticity, eventually contributing to memory impairment. Therefore, hippocampal synaptic plasticity has become the focus of investigations of the mechanism of memory impairment and exploration of intervention methods.

Acupuncture was first recorded in the *Yellow Emperor's Classic of Internal Medicine* and has now been used for thousands of years. Electroacupuncture (EA) and manual acupuncture (MA) are two forms of acupuncture, and both are widely used in clinical settings. The therapeutic effects of acupuncture in certain diseases, including gastrointestinal diseases [12, 13] and neurodegenerative diseases [14, 15], as well as effects in anesthesia and analgesia have been recognized. According to traditional Chinese medicine (TCM) theory, both AD and VCD belong to the category of dementia. Physical weakness leads to the loss of brain nutrition and function, eventually causing dementia and cognitive dysfunction. Acupuncture can refresh the mind, dredge the meridians, restore body energy and brain function, and show good clinical effects on AD and VCDs [16].

Acupuncture treatment is especially useful for AD and VCD since it can effectively prevent memory impairment, thereby improving patients' quality of life [17, 18]. The mechanisms underlying the effects of acupuncture on memory can be explored from the perspective of hippocampal synaptic plasticity. Acupuncture can change the structural characteristics and functionality of synaptic plasticity by modulating synaptic proteins, inhibiting inflammatory responses in neural pathways, etc. This review analyzes the selection of acupoints in the treatment of memory impairment and updates the current understanding of the action mechanisms underlying acupuncture. To complete this review, we extensively searched the English literature included in the PubMed and Web of Science databases and the Chinese literature in the China national knowledge infrastructure (CNKI) database over the last five years (2017-2022) with the search formula (AD or vascular cognitive disorders or cognitive impairment) and (acupuncture or electroacupuncture). The PubMed database search retrieved 481 studies; the Web of Science database search retrieved 255 studies; and the CNKI database search retrieved 1377 studies. The retrieved studies were screened to identify clinical trials and basic research studies with clear methods and results. We also discussed the potential strategies to improve the effect of acupuncture. Additionally, the effects of acupuncture on synaptic plasticity through regulation of the vascular–glia–neuron unit were further discussed.

## 2. Acupuncture Intervention for Memory Impairment in AD and VCDs

A preceding systematic review showed that the most frequent acupoints used in the treatment of AD are “Zusanli” (ST36), “Sishencong” (EX-HN1), “Baihui” (GV20), “Sanyinjiao” (SP6), “Neiguan” (PC6), “Shenting” (DU24), “Shenmen” (HT7), “Taixi” (KI3), “Hegu” (LI4), and “Dazhong” (KD4) [19]. Among them, “Baihui” (GV20) and “Shenting” (DU24) are included in the Governor Vessel, which belongs to the kidney collateral. Stimulation of the Governor Vessel has been suggested to replenish the kidney and increase the brain marrow, thereby improving cognitive symptoms. Acupuncture of the Governor Vessel can activate brain functional areas such as the hippocampus and improve synaptic plasticity [20, 21]. According to a meta-analysis of randomized controlled trials, acupuncture-related treatments lasting for at least 6 weeks can effectively improve the cognitive function of AD patients [22]. A systematic review also suggested that acupuncture was a promising complementary treatment for AD [23]. Acupuncture plus drug therapy (donepezil hydrochloride, nimodipine, or yizhijiannao) was especially shown to have more beneficial effects for AD patients in comparison with application of the drug alone [24]. Since acupuncture treatments in AD have been systematically reviewed or analyzed, we will not discuss them further here.

A number of studies have consistently shown that the Governor Vessel is also the most frequently used meridian for the treatment of VCDs, and “Baihui” (GV20) is the most frequently used acupoint. Yang meridians are the main acupoints in acupoint compatibility, but “Taixi” (KI3), “Sanyinjiao” (SP6), and other acupoints of the foot shaoyin kidney meridians and foot taiyin spleen meridians are also commonly used. These compatibility rules fully reflect the overall idea of acupuncture in the treatment of VCDs. In a randomized controlled clinical trial on acupuncture treatment of VCD [25], the researchers selected “Baihui” (GV20), “Yintang” (GV29), “Shenting” (GV24), “Shuigou” (GV26), “Naohu” (GV17), “Sishencong” (EX-HN1), and bilateral “Fengchi” (GB20), “Shenmen” (HT7), and “Sanyinjiao” (SP6) and other points, emphasizing that the Governor Vessel was the only meridian that directly entered the brain; thus, it was closely related to brain function and performed the functions of improving cognition and regulating mind. The combination of head and neck acupoints such as “Baihui” (GV20), “Sishencong” (EX-HN1), and “Fengchi” (GB20) can play a role in clearing the head, opening the body, and waking the mind. The compatibility of “Shenmen” (HT7) and “Sanyinjiao” (SP6) and other distal acupoints such as the heart meridian and spleen meridian can enhance the improvement of cognitive function by regulating the mind and supplementing Qi and blood. With the recent advancements in resting functional magnetic resonance imaging, positron emission tomography, and other brain functional imaging technologies, the improvement in cognitive function by acupuncture at acupoints such as “Baihui” (GV20), “Shenmen” (HT7), “Zusanli” (ST36), “Neiguan” (PC6), and “Taixi” (KI3) has been further verified [26]. Kim et al. demonstrated that treatment at “Fengchi” (GB20) and other Governor Vessel acupoints could regulate cerebrovascular compliance and improve cerebral blood flowing [27].

## 3. Potential Mechanisms Underlying the Effects of Acupuncture on Memory Impairment

### 3.1. Acupuncture Regulates Synaptic Protein Expression

**Figure 1:**
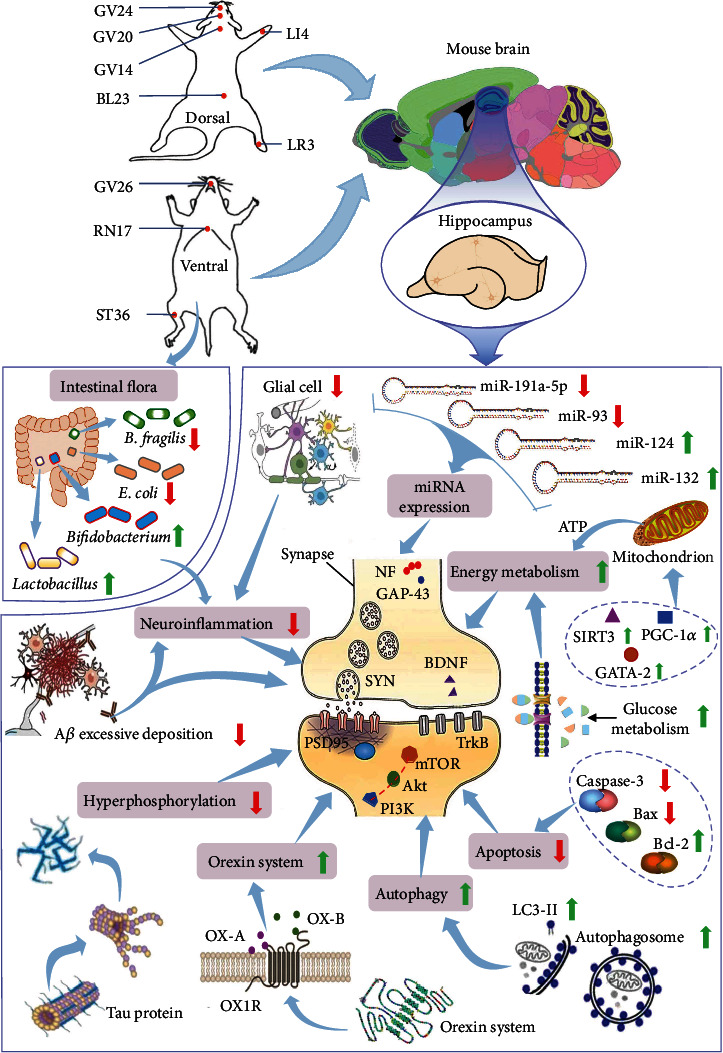
Potential mechanisms underlying the effects of acupuncture on hippocampal synaptic plasticity. The action mechanisms of acupuncture are closely related to the repair of hippocampal synaptic plasticity. Focusing on hippocampal synaptic plasticity, we reviewed the mechanisms underlying the effects of acupuncture on memory impairments through regulation of synaptic proteins, AD characteristic proteins, intestinal microbiota, neuroinflammation, microRNA expression, orexin system, energy metabolism, etc. The findings suggested that hippocampal synaptic plasticity may be common as well as the core link of the above mechanisms.

Synaptophysin (SYN), a specific marker of vesicle protein, is closely related to synaptic structure and function, and directly participates in synaptic formation [28, 29]. Various studies related to CI have shown that SYN is regulated by multiple protein signaling pathways. Xu et al. [30] found that inhibition of the mammalian target of rapamycin (mTOR)/nuclear factor kappa-B (NF-*κ*B) signaling pathway in a mouse model of diabetic encephalopathy resulted in improved cognitive performance and elevated levels of SYN to regulate synaptic plasticity. Activation of the N-methyl-d-aspartate receptor (NMDAR)-cAMP response element binding protein (CREB)-brain-derived neurotrophic factor (BDNF) pathway was also shown to promote SYN expression and improve memory impairment in senescence-accelerated mouse prone 8 (SAMP8) mice [31]. Similarly, BDNF overexpression in 5× familial AD (5-FAD) mice promoted the recovery of SYN and postsynaptic density protein-95 (PSD-95) as well as the number of presynaptic vesicles at excitatory synapses, enhanced long-term potentiation (LTP), and thus improved memory impairment [32]. On the other hand, a study by Li et al. demonstrated that Chst14/D4st1-deficient (Chst14^−/−^) mice showed impaired memory function, primarily due to reduced expression of synapse-associated proteins, including SYN [33]. Thus, activation of SYN expression levels by different pathways or methods in animal models of CI can affect synaptic plasticity and improve memory. Xie et al. [34] selected representative acupoints of the pericardium meridian and the lung meridian, and reported that acupuncture improved the expression of SYN with meridian specificity and regulated synaptic plasticity after cerebral ischemia injury **(**Figure 1**)**. As a sign of synaptogenesis [35], reduction of synaptic proteins in neurons of the cerebral cortex can lead to cognitive dysfunction. Zhao et al. stimulated “Danzhong” (CV17), “Zhongwan” (CV12), “Qihai” (CV6), bilateral “Xuehai” (SP10), and “Zusanli” (ST36) by sanjiao acupuncture and found that sanjiao acupuncture increased SYN expression, promoted neural regeneration and synaptogenesis, repaired damaged neurons, and improved memory impairment in SAMP8 mice [36].

PSD-95 is the most abundant scaffold protein in the synaptic dense part, modulating the postsynaptic response to glutamate release by regulating the anchoring of glutamate receptors. PSD-95 participates in the connection and formation of synapses, maintaining synaptic plasticity [37, 38]. Xu et al. [39] found that memory impairment in APP/PS1 mice was associated with impaired LTP and reduced NMDAR-mediated spontaneous excitatory postsynaptic currents, along with shorter hippocampal pyramidal neuron dendrites, reduced crossover points, and reduced spine density. Thus, the molecular mechanisms underlying the behavioral and neuropathological changes are associated with reduced levels of the NMDAR subunit and PSD-95 expression. Miaomiao et al. [40] found that EA could upregulate the expression of PSD-95 in the hippocampus and cortex of SAMP8 mice, and that early intervention with acupuncture could produce optimal therapeutic effects. Yang et al. [41] also suggested that EA at “Baihui” (GV20) and “Yongquan” (KI1) could promote the expression of PSD-95 and other synaptic proteins, reduce the deposition of A*β*, and improve synaptic plasticity of APP/PS1 mice. Similarly, in the study by Li et al. [42], EA stimulation of “Baihui” (GV20), “Dazhui” (GV14), and “Shenshu” (BL23) increased the expression level of PSD-95 in the hippocampal CA1 region of SAMP8 mice, improved synaptic ultrastructure, and reversed the learning and memory impairment.

Growth-associated protein-43 (GAP-43) is a specific phosphoprotein widely present in neuronal axons, and is a molecular marker of synaptic growth, development, and regeneration [43] **(**Figure 1**)**. 1-Methyl-4-phenyl-1,2,3,6-tetrahydropyridine (MPTP) induced dopaminergic neuronal damage in SAMP8 mice and learning and memory impairment, whereas environment enrichment directly increased GAP-43 expression in the substantia nigra of mice to protect dopaminergic neurons from MPTP injury [44]. Similarly, 1,4-butanediol is toxic to the nervous system and can reduce the expression of proteins such as GAP-43 through the ERK1/2-CREB-BDNF signaling pathway, affecting synaptic plasticity and inducing learning and memory impairments [45]. Thus, enhancement of neuronal synaptic plasticity by promoting GAP-43 expression levels is beneficial. Wang et al. [46] found that EA at “Baihui” (GV20) and “Shenshu” (BL23) could effectively regulate the synaptic structure and increase the activity of GAP-43, thereby reducing synaptic deficits, enhancing synaptic plasticity, and improving the learning and memory of *β*-amyloid protein 1-42 (A*β*1-42)-treated rats.

Neurofilament protein (NF) is a basic component of axons. NF is intertwined with itself and other proteins to form a structure that can transmit energy and signals. Therefore, upon brain tissue damage, the body produces various repair signals to stimulate related repair factors, including NF to repair neurons **(**Figure 1**)**. Thus, increased NF expression can facilitate neuron repair [47]. The neurofilament light chain (NEFL), a subunit of NF, is produced by both *nefla* and *neflb* genes in zebrafish. Since *neflb* knockdown resulted in significant apoptosis of neurons in the zebrafish brain and spinal cord, neuronal damage in zebrafish and these abnormalities could be reduced by promoting *neflb* expression [48]. Chao et al. [49] reported that EA at “Baihui” (GV20) and “Fengfu” (GV16) effectively increased the expression of NF and improved the memory of VCD rats.

The neurotrophin (NT) family is composed of many nutritional factors, including nerve growth factor (NGF), BDNF, NT3, NT4, and glial cell-derived neurotrophic factor. BDNF is the most abundant neurotrophic factor in the brain that regulates neuronal plasticity and promotes the development of neurons [50–52]. Activation of BDNF and TrkB has been shown to be required for synaptic aggregation. Mature BDNF maintains synaptic aggregation, whereas proBDNF downregulates asynchronous synapses, and NMDAR activation mediates asynchronous inhibition of proBDNF signaling. Thus, the push-pull plasticity mechanism of BDNF and proBDNF may regulate synaptic clustering [53]. CI is the main symptom in stroke and ischemia-reperfusion injury [54]. Zhen-yao et al. [55] reported that acupuncture combined with cognitive rehabilitation therapy effectively improved the CI of poststroke patients by upregulating BDNF. Lin et al. stimulated “Shenting” (DU24) and “Baihui” (DU20) by EA, and showed improvements in memory impairment in rats with cerebral ischemia-reperfusion (I/R) injury, along with upregulation of the expression levels of BDNF and PSD-95; thus, the mechanism of action underlying the effects of EA is related to the improvement of BDNF-mediated synaptic plasticity in hippocampal neurons [56].

Neural signaling pathways play an important role in neural development by promoting nerve regeneration and participating in inflammatory responses, immune cell chemotaxis, and tissue repair [57]. Therefore, regulation of neural signaling pathways is a therapeutic target for various neurological diseases [58]. MPTP treatment has been shown to induce autophagy in dopaminergic neurons and reduce neuronal viability, while insulin-like growth factor-1 (IGF-1) can inhibit MPTP-induced dopaminergic neuronal damage through activation of the IGF-1 receptor/PI3K-Akt-mTOR signaling pathway and G-protein-coupled ER 1 [59]. Our team found that activation of the BDNF/TrkB signaling pathway and regulation of NMDAR and *α*-amino-3-hydroxy-5-methyl-4-isoxazole propionic acid receptor (AMPAR) expression could improve synaptic plasticity in neurons [9]. Thus, activation of signaling pathways such as PI3K-AKT-mTOR and BDNF/TrkB is an effective means to protect neurons. Li et al. [60] also demonstrated that MA at “Baihui” (GV20) and “Zusanli” (ST36) improved memory impairment in rats with VCDs through activation of the PI3K/Akt/mTOR signaling pathway. In a poststroke cognitive impairment (PSCI) study, Zheng et al. found that EA stimulation of “Yintang” (EX-HN3) and “Baihui” (GV20) improved memory in middle cerebral artery occlusion (MCAO) rats, and a mechanistic study suggested an association with activation of the BDNF/TrkB signaling pathway and restoration of NMDAR, AMPAR, and *γ*-aminobutyric acid type A receptor expression [61]. Li et al. [62] also showed that MA continuously activated the BDNF/TrkB pathway and regulated LTP, thereby improving memory impairment in rats with traumatic brain injury. Wang et al. [63] found that “olfactory three needle” at “Yintang” (GV29), bilateral “Yingxiang” (LI20) can activate the PI3K-AKT signaling pathway, upregulate the expression of PSD-95, SYN, and GAP43 proteins, improve synaptic plasticity in A*β*1-42-treated rats, reduce apoptosis, and alleviate memory impairment. In addition, the study by Hou et al. [64] showed that EA at “Baihui” (GV20), bilateral “Zusanli” (ST36) could improve the memory impairment in SAMP8 mice, and that its mechanism was related to activation of cAMP/PKA/CREB signaling pathway and enhancing synaptic plasticity of hippocampal neurons. Similarly, acupuncture at the corresponding acupoints can inhibit GSK3*β*/mTOR in d-galactose-treated rats [65] and the AMPK/eEF2K/eEF2 [66] and RhoA/ROCK signaling pathways in SAMP8 mice to promote synaptic plasticity, thereby improving memory impairment [67].

### 3.2. Acupuncture Downregulates Alzheimer's Disease-Related Proteins

Tau is a multifunctional cytoskeletal protein that plays an important role in neurite formation, maintenance of cytoskeletal integrity, and axonal transport. The accumulation of hyperphosphorylated tau can cause neurofibrillary tangles and damage hippocampal synaptic plasticity, thereby seriously affecting learning and memory [68, 69] (Figure 1). Ma et al. [70] reported that EA at “Shenshu” (BL23) and “Baihui” (GV20) could reduce the expression of cycle-dependent protein kinase 5 (CDK5), inhibit tau, and improve the morphological structure of the hippocampus, thereby improving memory of SAMP8 mice. Yang et al. [71] treated SAMP8 mice with EA at “Baihui” (GV20), “Dazhui” (GV14), and “Shenshu” (BL23) to explore the therapeutic effect of early EA intervention. The results showed that early EA intervention can inhibit phosphorylation of tau, improve the ultrastructure of neurons, increase the number of synapses, and improve learning and memory in mice. Moreover, EA can regulate the activity of glycogen synthase kinase-3*β* during the phosphorylation of tau protein, inhibit abnormal glucose metabolism and tau phosphorylation, and enhance the learning and memory of APP/PS1 mice [72].

*β*-Amyloid precursor protein (A*β*) shows neurotoxicity that can cause synaptic degeneration and result in memory impairment. EA has been reported to inhibit A*β* in the hippocampus to improve the learning and memory function of APP/PS1 mice [73]. Wang et al. [74] used EA with different frequencies (2 Hz and 50 Hz) to stimulate “Baihui” (GV20) and “Shenshu” (BL23) and found that high-frequency EA can enhance the expression of the NMDAR subunit gene, increase the number of hippocampal synapses, and reduce APP, A*β*1-40, and A*β*25-35 protein levels, thereby improving memory impairment. Yang et al. [41] investigated the therapeutic effects of EA on “Baihui” (GV20) and “Yongquan” (KI1) in APP/PS1 mice, and showed that EA reduced hippocampal A*β* levels and increased PSD-95 and SYN expression in mice, suggesting that EA reduces the neurotoxicity of A*β* and thereby improves synaptic plasticity. Ling-ge et al. [75] stimulated “Baihui” (GV20) and “Dazhui” (GV14) with different intensities of EA to investigate the effects on VCD rats. The results showed that high-intensity combined with high-frequency EA stimulation can effectively inhibit the expression of A*β*1-40 in the hippocampal CA1 region and reduce synaptic damage, thereby improving learning and memory (Figure 1).

### 3.3. Acupuncture Shows Its Effects by Promoting Autophagy and Inhibiting Neuronal Apoptosis

The occurrence of autophagy depends on the participation of a series of autophagy-related proteins (ATGs). Beclin-1 complex is the core substance that may be involved in the initiation of autophagy, and it participates in the formation of subsequent autophagosomes. Microtubule-associated protein1 light chain 3 (LC3) is a light chain protein that plays a key role in the process of autophagy and participates in the formation of autophagy lysosomes. Therefore, Beclin-1 and LC3 are key indicators of autophagy activation. Cerebral ischemia and hypoxia can induce neuronal autophagy, resulting in neuronal damage [76]. Autophagy regulates synaptic plasticity by controlling the excitation–inhibition balance of neurons, including the inhibition of the central regulators of neuronal protein synthesis such as mTOR and BDNF [77], which can inhibit the synthesis of *β*-amyloid and tau. Autophagy can also restore synaptic proteins such as SYN and PSD-95 and degrade postsynaptic receptors, and mitophagy plays an important role in the release of synaptic vesicles and synaptic transmission [78, 79]. Gao et al. [80] found that acupuncture stimulation at the head acupoints of “Temporal Three Needles” can have a benign activation effect on autophagy and, to a certain extent, promote the repair of damaged neurons and improve learning and memory in rats with fetal intrauterine distress.

Apoptosis is a genetically controlled programmed cell death underlying the neuronal damage during cerebral ischemia and hypoxia. The Bcl protein family plays a crucial role in regulating neuronal apoptosis. Because of their different roles in apoptosis, proteins of the Bcl family are divided into two categories, Bcl-2 and Bax [81, 82]. Dong-dong et al. [83] found that acupuncture treatment at “Baihui” (GV20) and “Shenting” (GV24) could ameliorate memory through regulation of Bcl-2/Bax-dependent apoptosis to prevent memory impairment in MCAO rats. Huang et al. [84] explored the effects of EA on AD by stimulating “Baihui” (GV20), “Dazhui” (GV14), and “Zusanli” (ST36) of A*β*1-40-treated rats. The results showed that EA could upregulate the expression of Bcl-2, downregulate the expression of Bax, and inhibit the apoptosis of hippocampal neurons. Zhang et al. [85] found that EA reduced the expression levels of A*β*, caspase-3, and Bax proteins in the hippocampus, inhibited hippocampal neuronal apoptosis, and improved learning and memory function in APP/PS1 mice by stimulating “Baihui” (GV20), “Fengfu” (GV16), and “Shenshu” (BL23). In addition, some studies have pointed out that EA can also reduce hippocampal neuron apoptosis and enhance the learning and memory ability of permanent bilateral common carotid artery occlusion (2-VO) rats and APP/PS1 mice by inhibiting the c-Jun N-terminal protein kinase signaling pathway [86, 87] **(**Figure 1**)**.

### 3.4. Acupuncture Reduces Neuroinflammation

Neuroglia, including microglia, astrocytes, and oligodendrocytes, are required for neuron development, synapse formation, and the functioning of the central nervous system. In addition, neuroglia also produces a variety of inflammatory factors, and an excessive inflammatory response can damage neurons and cause synaptic deficits. Simultaneously, neuroglia also stimulates gliosis and cause neurodegeneration [88, 89]. Microglia is the main effector cells of neuroinflammation, and they play dual roles in protecting neurons and causing neuronal damage. Astrocytes also play an important role in synaptogenesis and synaptic transmission [90, 91]. Xie et al. [92] used EA to stimulate “Baihui” (GV20) in AD rats to explore its regulatory effects on neuroglia. The results showed that EA could reduce interleukin-1*β* (IL-1*β*), tumor necrosis factor-*α* (TNF-*α*), IL-6, and NF-*κ*B levels, increase the levels of IL-4 and IL-10, and improve memory impairments in A*β*1-42-treated rats. Wang et al. [93] used EA to stimulate “Baihui + Shenshu” (GV20 + BL23) and “Baihui + Shenshu + Feishu” (GV20 + BL23 + BL13) in A*β*25-35-treated rats, and found that both acupuncture schemes could downregulate prefrontal cortex and hippocampal IL-1*β* and TNF-*α*, reduce inflammatory reaction, and improve memory impairment. Yang et al. [94] reported that stimulation at “Baihui” (GV20), “Zusanli” (ST36), and other acupoints could reduce the expression of the inflammatory factors IL-1*β* and IL-6 in the cerebral cortex of rats and improve learning and memory in VCD rats. Wang et al. [24] adopted the TCM syndrome differentiation idea of tonifying the kidney and strengthening the brain, and found that EA could reduce activation of microglia and astrocytes and inhibit the inflammatory response, thereby improving memory in SAMP8 mice. In addition, Qiu et al. [95] explored the role and mechanism of EA in the treatment of 2-VO rats. The results showed that acupuncture at “Baihui” (GV20), “Dazhui” (GV14), and “Shenshu” (BL23) could reduce the expression of NOD-like receptor protein 3 (NLRP3), inflammatory reactions, and neuronal damage in the hippocampal CA1 area. Similarly, one study pointed out that EA at “Baihui” (GV20) and “Shenting” (GV24) can inhibit the activation of NLRP3 and prevent CI of presenilin1/2 conditional double-knockout mice [96].

As resident glial cells of the innate immune response, microglia and astrocytes contain many proinflammatory factors, IL-1*β*, tumor cytokines (TNF), and NF-*κ*B, etc. Activated microglia show high expression levels of proinflammatory cytokines, and coactivation of cytotoxicity leads to loss and decline of synaptic proteins [97]. IL-1*β* and TNF can pass through AMPAR and NMDAR, induce the occurrence of LTP, change synaptic number density, and promote the expression of synaptic proteins. Simultaneously, activated microglia mediates the deposition of A*β* [98], and the excessive deposition of A*β* also leads to neurotoxicity and affects synaptic plasticity. To this end, procytokines or cytokines could also be targets for acupuncture for regulation of synaptic plasticity.

### 3.5. Acupuncture Modulates the Orexin System

Neurons in the lateral hypothalamic region produce orexin, which has two subtypes: orexin A (OXA) and orexin B (OXB) [99]. Orexin neurons can project into the hippocampus and the medulla oblongata, and are involved in the regulation of synaptic plasticity and learning and memory. Orexin-1 receptor (OX1R) is a G protein receptor that can be activated by OXA and OXB, and the combination of the two can activate neurons in the CA1 region [100]. Liu et al. [101] used EA at “Baihui” (GV20), “Fengfu” (GV16), “Dazhui” (GV14), and “Shenting” (GV24), and the results showed that EA had a protective effect on neuronal damage in rats with chronic cerebral ischemia and promoted OX1R expression in the hippocampus. Hou et al. [64] reported that EA stimulation could improve memory impairment and inhibited OXA in cerebrospinal fluid by regulating the related proteins and neurotransmitters in a cAMP/PKA/CREB pathway-dependent manner.

OX1R and OX2R are G protein-coupled receptors and are widely distributed in the central nervous system. Orexin neurons can project into the hippocampus, causing an increase in intracellular calcium concentration, and regulate the LTP [102, 103]. Orexin can also release the excitatory neurotransmitter glutamate to transmit information and has shown some immune-protective effects on hippocampal neurons. In addition, microglia can also regulate orexin receptors upon inflammatory response [104].

### 3.6. Acupuncture Modulates Synaptic Proteins by Mediating microRNA Expression

microRNA (miRNA) can regulate the local synthesis of synaptic proteins and participate in the process of learning and memory [105, 106]. Liu et al. [107] demonstrated that EA at “Baihui” (GV20) and “Shenting” (GV24) improved learning and memory during the recovery stage of ischemic stroke. The mechanisms underlying these effects were related to miR-134-mediated LIM kinase 1, which regulated hippocampal synaptic plasticity. Zhou et al. [108] found that EA can improve neuronal damage and regulate synaptic plasticity in rats with cerebral ischemia, and that its improvement mechanism is related to downregulation of miR-191a-5p, targeting of neuronal calcium sensor 1 (NCS-1), BDNF, and GAP43, and protection of neurons. Using a cerebral ischemia-reperfusion rat model, Pan et al. [109] found that acupuncture at “Baihui” (GV20) and “Shenting” (GV24) could improve learning and memory by mediating miR-664-3p. Wang et al. [110] reported that acupuncture could reduce neuroinflammation and improve memory impairment by inhibiting miR-93 expression in rats with VCD. Liu et al. [111] explored the effects and mechanism of EA on neurological function in ischemic stroke, and found that EA can promote nerve regeneration and brain repair and reduce cognitive dysfunction by upregulating the expression of serum miR-124 and hippocampal miR-132.

miRNAs play important roles in neurogenesis, cell migration, neuron maturation, dendritic branching, axon regeneration, synapse development, and synaptic transmission [112] (Figure 1). miRNAs can also directly participate in the regulation of local synaptic protein synthesis, and have effects on neurotransmission and synaptic activity [109]. miRNAs regulate target genes and signals through translation, transcription, degradation, etc., thereby regulating the number of dendritic spines in hippocampal neurons, the release of neurotransmitters, and the synaptic structure [105].

### 3.7. Acupuncture Modulates Gut Flora

Aging and neurodegenerative diseases are also important factors influencing changes in the diversity of intestinal flora. The gut microbiota can affect cognitive function through the gut-brain axis. Impairment of gut microbiota produces large amounts of lipopolysaccharide, a proinflammatory neurotoxic substance. Lipopolysaccharide in the gut can enter the brain through the physiological barrier, induce neuroinflammatory responses, and lead to cognitive dysfunction [113, 114] **(**Figure 1**)**. He et al. [115] investigated the therapeutic effects of EA on AD. The results showed that EA stimulation at “Baihui” (GV20) and “Zusanli” (ST36) could increase the DNA abundance of *Lactobacillus* and *Bifidobacterium*, reduce the DNA abundance of *E. coli* and *B. fragilis*, and restore the learning and memory of d-galactose-treated rats by regulating intestinal flora, inhibiting the level of lipopolysaccharide. Dong-mei et al. [116] performed EA at “Baihui” (GV20), “Zusanli” (ST36), “Dachangshu” (BL25), and other acupoints and found that EA could improve memory impairments in APP/PS1 mice by regulating the diversity of intestinal flora and inflammation in mice. Yang et al. [117] found that acupuncture at “Baihui” (GV20), “Hegu” (LI4), “Feishu” (BL13), “Pishu” (BL20), “Shenshu” (BL23), “Zusanli” (ST36), and “Sanyinjiao” (SP6) influenced mouse microbiota and metabolites in APP/PS1 transgenic mice. EA at “Baihui” (GV20) and “Yintang” (GV29) could improve learning and memory in SAMP8 mice by balancing the quantity and composition of gut microbiome, especially the relative abundance in deltaproteobacteria and epsilonproteobacteria [118]. Chen et al. [119] reported that EA at “Baihui” (GV20), “Dazhui” (GV14), “Shenshu” (BL23), and “Zusanli” (ST36) could improve cognitive dysfunction in VCD model rats, which may be related to its function in regulating the imbalance of intestinal microbiota and thereby inhibiting peripheral inflammatory factors.

The intestinal flora can not only affect the neuroglia and neurons of the human central nervous system, but can also produce a variety of metabolites such as dopamine and lipopolysaccharide and neurotransmitters such as serotonin, which can also transmit information through the vagus nerves, induce the occurrence of LTP, and affect synaptic plasticity [120]. Gut microbes can regulate the transmission of brain signals through the immune, endocrine, metabolic systems, etc. For example, when A*β* is excessively deposited, the intestinal flora can stimulate the immune system of the brain to respond to the inflammatory response of A*β* [121]. Gut microbiota can also inhibit microglial activation and attenuate microglia-induced oxidative stress and proinflammatory cytokines. On the other hand, dysbiosis of gut microbiota reduces BDNF expression in the hippocampus and cerebral cortex, affecting synaptic plasticity in the hippocampus and brain function [122].

### 3.8. Acupuncture Modulates Energy Metabolism

Adenosine triphosphate (ATP) produced by mitochondria is the most important energy source for high-energy-demanding organs and plays an important role in maintaining the functional integrity of brain neurons, especially hippocampal neurons. Therefore, mitochondrial dysfunction will affect the release and transport of neurotransmitters, which in turn affects the structure and function of the brain, resulting in impairment of cognitive function [123]. Wenqiang et al. [124] proved that acupuncture could upregulate the expression of sirtuin3 (SIRT30) and GATA factor 2, improve mitochondrial function, regulate energy metabolism, and improve the learning and memory function of SAMP8 mice. Zhang et al. [125] observed that acupuncture in SAMP8 mice can increase the expression of SIRT3 and peroxisome proliferator-activated receptor gamma coactivator-1*α* in the hippocampus, improve mitochondrial energy metabolism, and thereby improve learning and memory.

In the central nervous system, glucose and lactate must be transported across the membrane by means of transporters such as the glucose transporter (GLUT) and the monocarboxylic acid transporter (MCT). The levels of these transporters can directly affect the substrate uptake efficiency of neurons. Reduction of GLUT1 will cause obstacles in A*β* clearance and lead to hyperphosphorylation of tau. The subsequent imbalance in transporters will also affect neuronal functional activities related to learning and memory [126]. Liu et al. [127] found that EA can increase the expression of GLUT1 and GLUT3 in the hippocampus and cortex and improve the learning and memory of APP/PS1 mice by regulating glucose metabolism. Junyan et al. [128] also proved that acupuncture at “Baihui” (GV20) and “Shuigou” (GV26) could increase the expression of GLUTs in the hippocampus of rats and reduce neuronal damage by promoting glucose metabolism. EA stimulation at “Baihui” (GV20), “Yintang” (GV29), and “Shuigou” (GV26) in APP/PS1 mice could increase the frontal lobe glucose uptake rate, indicating enhancement of glucose metabolism in the brain and improvement in the cognitive ability of mice after EA [129]. Similarly, Cao et al. [130] studied the effects and mechanisms of EA on AD mice. The results showed that EA at “Baihui” (GV20), “Yintang” (GV29), and “Shuigou” (GV26) could increase hippocampal glucose metabolism, improve the learning and memory ability of APP/PS1 mice, and yield slightly better therapeutic effects than those of donepezil. In addition, EA stimulation at “Quchi” (LI11) and “Zusanli” (ST36) in rats with cerebral ischemia and stroke promotes glucose metabolism in the caudate putamen (CPu), motor cortex (MCTX), and somatosensory cortex (SCTX), indicating that EA could enhance neural activities in CPu, MCTX, and SCTX regions, which is beneficial for the survival of neurons and the improvement of cognitive function [131].

Mitochondrial dysfunction and reduced ATP production can affect the occurrence of LTP, which will lead to reduced release of synaptic vesicles, while normal ATP concentration will sustain the release of neurotransmitters. The transmission and regulation of synapses also affect the structural and functional plasticity of synapses [132]. In addition, insulin growth factor, glucose transporter, etc. also play important roles in the metabolism and regulation of neuronal synapses, transducing signals by activating specific neural signaling pathways and providing sufficient energy substrates for the metabolism of hippocampal neurons to ensure normal occurrence of LTP and improving learning and memory by modulating synaptic plasticity [133] (Table 1).

## 4. How to Improve the Effect of Acupuncture

Acupoint selection for the treatment of memory impairment is based on the symptoms as well as the underlying disease. The acupoints located on the head are the most frequently selected. Among them, “Baihui” (GV20) is one of the acupoints at Governor Vessel and the most frequently used in clinical practice and experimental studies. From the perspective of TCM, neurodegenerative diseases with CI are all considered to be diseases of the brain. The experimental evidence described above suggests that acupuncture at “Baihui” (GV20) can facilitate the treatment of encephalopathy. In addition to “Baihui” (GV20), other acupoints are also selected on the basis of the disease characteristics and the outcomes of different types of TCM syndrome differentiation. However, there is no unified clinical treatment regime for this treatment. Therefore, acupoint selection could be standardized, and the treatment of the main symptoms and improvement of cognition can be selected cooperatively. For example, when treating CI of Parkinson's disease (PD), we should consider selecting acupoints for PD, as well as for CI.

Acupoint specificity is an important factor in determining the efficacy of acupuncture in treatment of diseases, including memory impairment. Data mining techniques have been extensively used to analyze the rules of acupoint selection. For mild CI, modern physicians mainly select acupoints locally, in combination with dialectics; yang meridians are the most popular meridians, and “Baihui” (GV20), which is the sea governor of yang meridians, is the most popular [134, 135]. The principles of upper and lower acupoints and distant and near acupoints are important aspects of acupoint compatibility. For example, “Baihui” (GV20) is mainly selected to be compatible with kidney-tonifying and spleen-strengthening acupoints.

In comparison with MA, EA can continuously stimulate acupoints, thereby increasing the efficacy of acupuncture. Clinical research shows that under the same treatment conditions, the therapeutic effect of EA on stroke is better than that of MA [136, 137], and EA treatment also yields better results in the treatment of other neurological diseases. Shi et al. [138] indicated that both EA and MA can fight against inflammation and pain, but EA showed a better effect. In a comparison of the efficacy of MA and EA in combination with selective serotonin reuptake inhibitors (SSRIs) in the treatment of depression, the therapeutic effect of EA was more significant (dispersive dense wave, 2 Hz low-frequency wave and 15 Hz high-frequency wave alternate) [139]. However, another study suggested that MA and EA showed similar effects [140, 141]. On the basis of comparisons of EA and MA in the treatment of encephalopathy and neurological diseases, we believe that while the effectiveness of EA in comparison with MA in the treatment of different diseases is unclear, at specific wave frequencies, the effectiveness of EA is better than that of MA.

In addition to the comparisons between MA and EA, the stimulation intensity and frequency of EA are also important for disease treatment. Jiang et al. [142] compared the therapeutic effects of EA on 4-vessels occlusion (4-VO) rats by observing the effects of stimulation at “Baihui” (GV20) and “Dazhui” (GV14) with different EA intensities, and found that EA could inhibit the A*β*1-40 miRNA expression of the hippocampal CA1 region, and high-intensity EA can better improve the learning and memory function than low-intensity EA. In addition, EA at a frequency of 2 Hz (low-frequency) and the 100 Hz (high-frequency) exchange output demonstrated a superior effect on opening the blood–brain barrier (BBB) in comparison with other fixed frequencies [143]. A fixed frequency of 2 or 100 Hz may show easy adaptability and a relatively continuous and stable stimulation, which may reduce the effect of EA [143]. Therefore, 2/100 Hz EA was applied to the GV20 and GV26 acupoints for 40 min, effectively increasing BBB permeability in rats [144]. However, for hippocampal synaptic transmission, high-frequency EA could demonstrate a better effect in rats with Alzheimer's disease [145]. Wang et al. [46] investigated the therapeutic effects and mechanisms of EA of “Baihui” (GV20) and “Shenshu” (BL23) at different frequencies (50, 30, and 2 Hz) in AD rats. The results showed that EA could downregulate GSK-3*β* and upregulate GAP-43 levels in the hippocampus, and the effect of EA at 50 Hz was better than at 30 Hz and 2 Hz in improving learning and memory function and repairing synaptic damage in AD rats.

**Figure 2:**
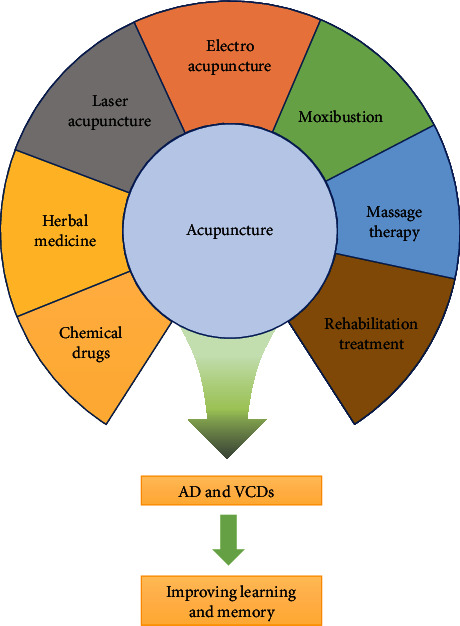
Strategies to improve the effect of acupuncture on memory impairment. EA and laser acupuncture, massage and rehabilitation therapy, combined application of acupuncture with herbal medicine, and chemical drugs or moxibustion could promote the effect of acupuncture on memory impairment in AD and VCDs.

Coapplication of drugs has also been attempted to improve the effects of acupuncture on memory impairment. Yang et al. [146] explored the effects and mechanism of EA stimulation at “Baihui” (GV20) and “Yintang” (EX-HN3) in combination with donepezil for the treatment of AD. The results showed that EA could enhance the effects of donepezil in improving learning and memory function in AD and promote donepezil to transport A*β* through the BBB by regulating the expression of matrix metalloproteinase-9, low density lipoprotein receptor-related protein-1, and Pgp. Huang et al. [84, 147] used EA for acupuncture at the “Baihui” (GV 20), “Dazhui” (GV 14), and “Zusanli” (ST36) acupoints in combination with gastrodin to treat learning and memory impairments in AD rats. The results showed that EA or gastrodin could improve the cognition of AD rats, upregulate SIRT1, Bcl-2, and PGC-1*ɑ*, inhibit the expression of Bax, and protect hippocampal neurons, but the therapeutic effect of EA in combination with gastrodin is better than that of EA or gastrodin alone (Figure 2). Interestingly, laser acupuncture improved cerebral ischemia-induced cognitive deficits and modulated the expression of creb, bdnf, bcl-2, and bax genes, exerting neuroprotective effects [148]. In addition, some physical therapies such as transcranial magnetic stimulation, moxibustion, massage, and rehabilitation therapy can also improve the effectiveness of acupuncture and require attention [149].

## 5. The Systemic Mechanisms Underlying the Effects of Acupuncture on CI Require Clarification

Acupuncture can modulate the structure and function of synaptic plasticity by regulating the expression of various synaptic proteins such as SYN, GAP-43, and PSD-95. Acupuncture can also affect synaptic plasticity through other mechanisms such as miRNA, gut flora, and neural circuits, thereby improving learning and memory. However, the current studies on the overall mechanism of acupuncture in improving CI by regulating synaptic plasticity are still fragmented and confusing. Most researchers have only studied and elaborated small parts of action mechanisms, and many studies have focused on synaptic proteins. Without an understanding of causality, the effects of acupuncture as perceived by the body after piercing the skin and the pathways through which signals are transmitted to the brain to regulate hippocampal synaptic plasticity are difficult to clarify. Therefore, the author believes that the mechanisms underlying the regulation of synaptic plasticity by acupuncture and the improvement in CI should be comprehensively expounded from various aspects.

After penetration of the skin at the acupoint, the acupuncture needle produces local microtraumatic stimulation. The skin contains a complex distribution of nerves, hormones, related receptors, and various cells, and acupuncture can activate the microenvironment near the acupoint area. By stimulating the peripheral nerves and exosomes that can carry the effective active ingredients of acupuncture, signals are transmitted to the target organs, causing local nerve excitation, activating the functions of numerous cells, and releasing chemical substances such as important messengers in synaptic and neuronal transmission. These substances interact with and influence each other, causing local inflammatory responses and activating the expression of various related proteins and inflammatory factors [150] through the cholinergic anti-inflammatory pathway, hypothalamic-pituitary-adrenal axis (HPA axis) [151], brain-gut axis, and vagus-adrenal axis [152], etc. to the medulla oblongata and the brain. The signals are then integrated by the central nervous system and play some physiological roles through the signaling pathways to regulate the stability of the body's internal environment [153]. The effects of acupuncture on synaptic plasticity and CI improvement constitute a multitarget, multilevel, and complex network system, which cannot be described from a single perspective. Therefore, future studies on the effects of acupuncture in regulating synaptic plasticity and improving CI should focus more on systemic networks. For example, acupuncture at “Baihui” (GV20) can first stimulate some related cells and proteins, which transmit information to the medulla oblongata and brain through specific neural signal pathways, and finally act on the hippocampus to regulate CI.

## 6. Vascular–Glia–Neuron Unit Might Be a Critical Target for Acupuncture

**Figure 3:**
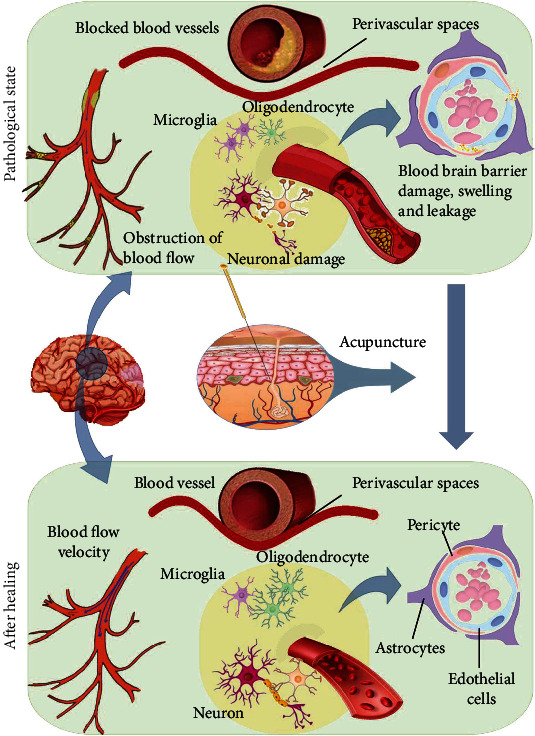
Vascular–glia–neuron units might be a critical target for acupuncture in the treatment of memory impairment. Acupuncture can upregulate vascular endothelial growth factor (VEGF), vascular endothelial growth factor receptor 2 (VEGFR2), basic fibroblast growth factor (bFGF), and CD34+, thereby promoting the proliferation of vascular endothelial cells, increasing local cerebral blood flow, activating multiple signaling pathways, and mediating vascular and nerve regeneration.

VCD is a clinical syndrome in which cerebral blood perfusion disorders are caused by cerebrovascular deficits, resulting in damage to local brain tissue cells, cognitive dysfunction, and even dementia [154]. Although the pathogenesis of AD is complex, vascular risk factors in midlife have been shown to be important causes of memory impairment in AD and are positively correlated with brain amyloid deposition [155]. Therefore, vascular CI caused by chronic cerebral hypoperfusion (CCH) is believed to be common in the occurrence and development of VCDs and AD, and is a common high-risk factor for cognitive decline [156]. The blood–brain barrier damage caused by cerebral ischemia and hypoxia can cause accumulation of toxic substances, production of inflammatory cytokines, and immune cell damage, which are early pathophysiological changes in many central nervous system diseases, including VCDs and AD [157]. Acupuncture can upregulate vascular endothelial growth factor (VEGF), vascular endothelial growth factor receptor 2 (VEGFR2), basic fibroblast growth factor (bFGF), and CD34^+^, thereby promoting the proliferation of vascular endothelial cells, increasing local cerebral blood flow, activating multiple signaling pathways, and mediating vascular and nerve regeneration [158]. EA stimulation of “Baihui” (GV20) and “Shuigou” (GV26) at a specific frequency has been reported to increase the permeability of BBB, providing a strategy for the treatment of nervous system diseases [143, 144]. Liu et al. [111] found that EA could regulate the expression of VEGF genes and promote angiogenesis in the ischemic cerebral cortex, which may play an important role in the improvement of CI in ischemic stroke. Similarly, Li et al. [159] also reported that EA can protect the neurovascular units of ischemic stroke rats and improve the symptoms of neurological deficits. Therefore, blood vessels and plasticity may also be important targets for acupuncture (Figure 3).

## 7. Conclusion

Acupuncture improves memory by regulating synaptic proteins, AD characteristic proteins, gut microbiota, neuroinflammation, miRNA, the orexin system, insulin receptors, and mitochondrial function and by other mechanisms. Regulation of synaptic plasticity may be a common and core link among these mechanisms. Although synaptic plasticity is the outcome of acupuncture interventions for CI, acupuncture may also stimulate a variety of different mechanisms and systems to interact with each other in interventions for different types of cognitive impairments, resulting in a cascade reaction to achieve the final effect.

## Figures and Tables

**Table 1:** Acupuncture interventions memory impairment in AD and VCDs.

Models	Treatment	Acupoints	Parameters
2-VO rats	MA	“Baihui” (GV20), “Zusanli” (ST36) [60]	3 mm, 1 time/d, continuous acupuncture for 6 days, rest for 1 day, 2 weeks
2-VO rats	EA	“Baihui” (GV20), “Dazhui” (GV14), “Housanli” “Geshu” (BL17) [86]	“Dazhui”: Straight stab, 2 ~ 3 mm. “Baihui”: Forward stab, 2 ~ 4 mm. “Geshu”: Straight stab, 3 ~ 5 mm. “Housanli”: Straight stab, 5 ~ 6 mm. Rarefaction, 2 Hz, 2 mA, 10 min/d, 14 days.
2-VO rats	EA	“Baihui” (GV20), “Shenshu” (BL20), “Dazhui” (GV14) [95]	“Dazhui”: Straight stab, 5 mm. “Baihui”: Oblique stab, 2 mm. “Shenshu”: Straight stab, 3 mm. Dilatational wave, 10 Hz/50 Hz, 1 mA, 30 min/d, 4 weeks.
2-VO rats	MA	“Baihui” (GV20), “Zusanli” (ST36) [110]	4 mm, 10 min/d, 2weeks. The rats rested once after six times of treatment
2-VO rats	EA	“Baihui” (GV20), “Shenshu” (BL23), “Dazhui” (GV14), “Zusanli” (ST36) [119]	“Baihui” oblique needling 2 mm, “Dazhui” and “Zusanli” direct needling 5 mm, “Shenshu” direct needling 3 mm, dilatational wave (10 Hz/50 Hz), 30 min/d, 4 weeks
4-VO rats	EA	“Baihui” (GV20), “Dazhui” (GV14) [75]	2 Hz/15 Hz, 0.5 mA, 1.5 mA, 30 min/d, 10 times
MCAO rats	Combination of acupuncture and medicine	“Baihui” (GV 20), “Shenting” (GV24) [83]	40 min/d, 8 weeks.
2-VO rats	MA	“Baihui” (GV20), “Zusanli” (ST36) [94]	Row twist compensation method (<90°, >120 times/min, 30 s), once a day, 6 days of treatment, 1 day of rest, a total treatment of 12 times
SAMP8 mice	MA	“Danzhong” (CV17), “Zhongwan” (CV12), “Qihai” (CV6) [36]	Twisting reinforcing manipulation method, 1 time/d, 15 d, and suspended only on day 7
APP/PS1 mice	EA	“Baihui” (GV20), “Yongquan” (KI1) [41]	2 ~ 3 mm, “Baihui”: Flat stab, “Yongquan”: Straight stab, density wave, 1 Hz/50 Hz, 0.5 mA, 15 min/d, 3 times/week, 6 weeks
SAMP8 mice	EA	“Baihui” (GV20), “Zusanli” (ST36) [64]	3 mm, 1 mA, 10 Hz, 30 min/d, 14 days
D-galactose-treated rats	EA	“Baihui” (GV20), “Shenshu” (BL23) [65]	“Baihui”: Inserted 15°obliquely, 2 mm. “Shenshu”: Inserted perpendicularly, 4-6 mm. 50 Hz, 20 min/d, 6 d/week, 8 weeks
SAMP8 mice	EA	“Baihui” (GV20), “Shenshu” (BL23), “Dazhui” (GV14) [66]	“Baihui”: Inserted horizontally downward, “Dazhui” and “Shenshu”: Perpendicularly, 2 Hz, 1 mA, 20 min/d, 8 days, and 2 days of rest, 30 days
SAMP8 mice	MA	“Danzhong” (CV17), “Zhongwan” (CV12), “Qihai” (CV6), “Xuehai” (SP10), “Zusanli” (ST36) [67]	“Xuehai”: 2 ~ 5 mm, <90°, >120 times/min, 30 s. “Danzhong”, “Zhongwan”, “Qihai”, “Zusanli”: 2 ~ 3 mm, 90°, >120 times/min, 30 s. 1 time/d, 28 days
SAMP8 mice	EA	“Baihui” (GV20), “Dazhui” (GV14), “Shenshu” (BL23) [71]	“Baihui”: Oblique stab, 2 mm. “Dazhui”: Oblique stab, 2 ~ 3 mm. “Shenshu”: Oblique stab, 3-4 mm. Continuous wave, 2 Hz, 1.5 ~ 2 mA, 20 min/d, 8 d/course of treatment, 8 course of treatment, treatment interval 2 days
APP/PS1 mice	EA	“Baihui” (GV20), “Shuigou” (GV26), “Yintang” (GV29) [72]	“Baihui”: Inserted backward, “Yintang”: Inserted towards the tip of the nose, 5 mm, 2 Hz, 1 mA, 20 min, turn off the EA apparatus and a quick prick was delivered at “Shuigou”. Once every other day for 28 days
AD rat model	EA	“Baihui” (GV20), “Shenshu” (BL23) [74]	“Baihui”: Horizontal needling, 5 mm. “Shenshu”: Oblique stab, 5 mm. Continuous wave, 1 mA, 2 Hz, 50 Hz, 1 time/1d, 7 d/course of treatment, 2 course of treatment
APP/PS1 mice	EA	“Baihui” (GV20), “Yongquan” (KI1) [41]	2 ~ 3 mm, “Baihui”: Flat stab, “Yongquan”: Straight stab, density wave, 1 Hz/50 Hz, 0.5 mA, 15 min/d, 3 times/week, 6 weeks
A*β*1-40-treated rats	EA	“Baihui” (GV20), “Zusanli” (ST36), “Dazhui” (GV14) [84]	1 ~ 2 mA, 2 Hz, 30 min/d, 4 weeks
APP/PS1 mice	EA	“Baihui” (GV20), “Shenshu” (BL23), “Fengfu” (GV16) [85]	“Baihui”: Backward oblique stab, 2 mm. “Shenshu”: Straight stab, 2 mm. “Fengfu”: Backward and downward oblique stab, 2 mm. Intermittent wave, 0.2 ms, 10 Hz, 2 mA, 20 min/d, 6 d/week, 16 weeks
APP/PS1 mice	EA	“Baihui” (GV20), “Yintang” (GV29), “Shuigou” (GV26) [87]	“Shuigou”: Fast pricking. “Baihui”, “Yintang”: 1 mA, 1 Hz, 20 min. Performed once every other day, 4 weeks
A*β*1-42-treated rats	EA	“Baihui” (GV20) [92]	2 mm, 2 ~ 4 V, 20 mA, 20 Hz, 30 min, 6 d/week, 3 weeks
A*β*25-35-treated rats	EA	“Baihui” (GV20), “Shenshu” (BL23), “Feishu” (BL13) [93]	“Baihui”: Forward stab, 3 ~ 5 mm. “Shenshu”: Slightly inward oblique stab, 5 ~ 6 mm. “Feishu”: Slightly inward oblique stab, 5 mm. 2 Hz, 2 mA, 15 min/d, 10 days
SAMP8 mice	EA	“Baihui” (GV20), “Shenshu” (BL23), “Taixi” (KI3) [160]	0.5 cm, sparse wave, 2 Hz, 2 V, 0.6 mA, 15 min/d, 2 months
Presenilin 1/2 conditional double knockout mice	EA	“Baihui” (GV20), “Shenting” (GV24) [96]	4 mm, 2 Hz, 1 mA, 15 min, 5 d/week, 3 weeks
D-galactose-treated rats	EA	“Baihui” (GV20), “Zusanli” (ST36) [115]	“Baihui”: 15° oblique stab, 2 mm. “Zusanli”: Straight stab, 4 mm. Continuous wave, 50 Hz, 1 mA, 20 min/d, 8 weeks
APP/PS1 mice	MA	“Baihui” (GV20), “Hegu” (LI4), “Feishu” (BL13), “Pishu” (BL20), “Shenshu” (BL23), “Zusanli” (ST36), and “Sanyinjiao” (SP6) [117]	20 min/d, 5 times/week, 4 weeks
SAMP8 mice	EA	“Baihui” (GV20), “Yintang” (GV29) [118]	Transverse puncturing, 4 ~ 5 mm, sparse wave, 2 Hz, 2 V, 0.1 mA, 15 min/d
APP/PS1 mice	EA	“Baihui” (GV20) [127]	Disperse waves, 1 and 20 Hz, 30 min, 5 days/week, and 2 days rest for a period of 4 weeks
APP/PS1 mice	EA	“Baihui” (GV20), “Yintang” (GV29, “Shuigou” (GV26) [129]	“Baihui”: Upward oblique stab, 5 mm and “Yintang”: Downward oblique stab, 5 mm. 2 Hz, 1 mA. “Shuigou”: After the electroacupuncture, quickly prick. 20 min/d, 4 weeks.
APP/PS1 mice	EA	“Baihui” (GV20), “Yintang” (GV29, “Shuigou” (GV26) [130]	“Baihui”: Downward horizontal, 0.5 cm, “Yintang”: Upward horizontal, 0.5 cm, “Shuigou”: After the electroacupuncture, “Shuigou”: After the electroacupuncture, vertical pricking. 2 Hz, 2 V, 0.1 mA, 20 min/d, 15 days

Abbreviations: EA: electroacupuncture; MA: manual acupuncture; 2-VO: permanent bilateral common carotid artery occlusion; 4-VO: 4-vessels occlusion; MCAO: middle cerebral artery occlusion; SAMP8: senescence-accelerated mouse prone 8; APP/PS1: APPSwe/PS1deltaE9.
